# Factors Associated with Quality of Dying and Death in Korean Intensive Care Units: Perceptions of Nurses

**DOI:** 10.3390/healthcare9010040

**Published:** 2021-01-05

**Authors:** Haeyoung Lee, Seung-Hye Choi

**Affiliations:** 1 Red Cross College of Nursing, Chung-Ang University, Seoul 06974, Korea; im0202@cau.ac.kr; 2 College of Nursing, Gachon University, Incheon 21936, Korea

**Keywords:** critical care, intensive care unit, person-centered care, quality of death, quality of dying

## Abstract

The objective of this study was to investigate the factors affecting the quality of dying and death among terminally ill patients in an intensive care unit in Korea using a cross-sectional, online survey. A total of 300 nurses in the intensive care unit who had cared for a terminally ill patient for at least 48 h prior to death in the past six months were chosen to participate. The person-centered critical care nursing (PCCN) score and quality of dying and death (QODD) had a positive correlation. The QODD score increased when the consultation was conducted between the terminally ill patients and their doctors when CPR was not performed within 48 h of death, and when the PCCN score increased. The quality of death of patients is affected by whether they have sufficiently consulted with healthcare providers regarding their death and how much respect they receive. It is important for nurses to practice and improve patient-centered nursing care in order to ensure a good quality of death for terminally ill patients.

## 1. Introduction

Among the elderly aged 65 years and above, the mortality rate of those living at home with their family is 14.4%, while that of those admitted to medical institutions, including nursing facilities, is 77.1% [1]. In other words, most people die in medical institutions in South Korea. Due to become an aging society, the advancement of medical services, and various life support equipment, many patients are admitted to the intensive care unit (ICU) in Korea. Many terminally ill patients in the ICU cannot make decisions on their own treatment due to tracheal intubation or continuous use of sedatives [2]. Accordingly, healthcare providers who work in the ICU play an important role in providing care and comfort for patients facing their own death.

In Korea, only seven days are observed between the suspension of life-sustaining treatment and the death of patients [3]. As a result, the wishes of patients cannot be properly considered because their condition has already deteriorated when discussions on their desired care begin. A good quality death involves a dynamic and continuous process of striving to properly prepare and achieve the wishes of terminally ill patients through the interaction of the patient, the patient’s family, and healthcare providers. This allows patients to face death with dignity and control and ensure the family of terminally ill patients are prepared to accept their death with positive emotions [4]. In 1997, a healthcare provider was charged with homicide for discontinuing the use of a ventilator following a discharge request from the patient’s family, which led to a healthcare provider in South Korea adopting a passive attitude towards terminating life-sustaining treatment [5]. In 2008, however, a hospital was sued for rejecting a family’s request for a patient’s dignified death, increasing the need for legal guidelines on terminating life-sustaining treatment [5]. The “Act on Hospice and Palliative Care and Decisions on Life-Sustaining Treatment for Patients at the End of Life” were enacted in February 2018 in an effort to ensure patients’ human rights and dignity, but even now, two years later, the decision to defer or suspend the life-sustaining treatment of the patient is still made by their family 70% of the time [6].

Nurses who care for terminally ill patients have the responsibility to ensure that they have a graceful and comfortable death [7]. To fulfill this responsibility, it is important to recognize person-centered care as an element through which the patient-nurse relationship is perceived as providing dignified care [8]. Therefore, this study comprehensively investigated the factors that affect the quality of death of terminally ill patients in the ICU as evaluated by nurses (hereafter, “patient factors”) as well as factors including the extent of person-centered care provided by nurses (hereafter, “nurse factors”).

The study aimed to investigate the factors associated with the quality of dying and death among terminally ill patients in an intensive care unit in Korea as perceived by nurses.

## 2. Materials and Methods

### 2.1. Study Design

This study was designed as a cross-sectional survey.

### 2.2. Setting

This study used an online survey company to randomly select ICU nurses across Korea and distribute the research survey. Snowball sampling was then used to recruit participants from among those who voluntarily consented to participate.

### 2.3. Participants

The inclusion criteria were nurses who: had been working in the ICU for at least six months, caring for a terminally ill patient for at least 48 h prior to the patient’s death within the past six months and had a clear understanding of the purpose of the study and consented to participate. Nurses that had a family member or relative who passed away in the past six months were excluded. Using G*Power program 3.1.9.2, the number of participants required with an effect size (F) of 0.25, significance level (*p*) of 0.05, and statistical power (1-β) of 0.95 was determined to be 176 for a t-test, 84 for an ANOVA, and 53 for a regression analysis [9]. Considering a response rate of 60% to the online survey, 300 participants were recruited from May to August 2020.

**Figure 1:**
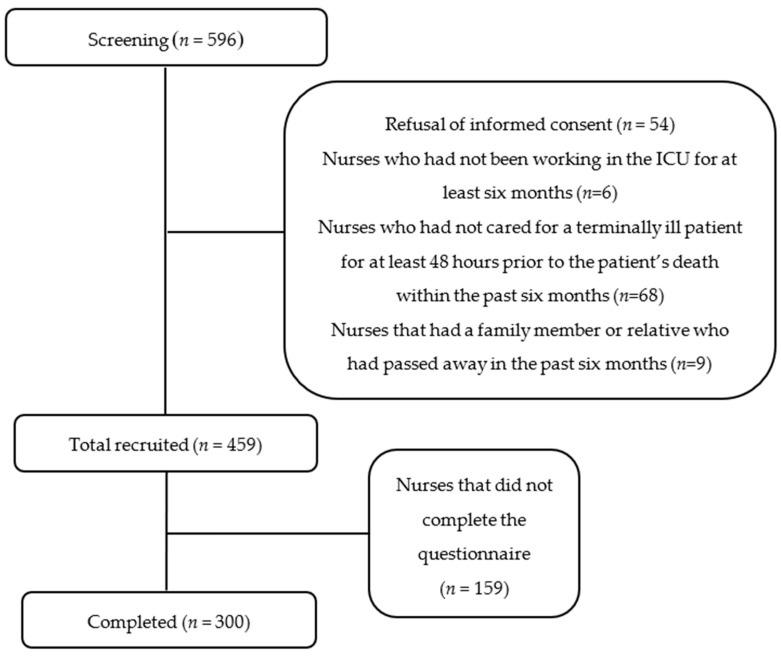
Recruitment flow chart.

In total, 596 nurses were selected as participants. However, 300 participants were recruited for the final analysis after excluding those individuals who did not provide consent (*n* = 54), those who had no experience of working in the ICU in the past six months (*n* = 6), those who had not cared for a terminally ill patient 48 h prior to death (*n* = 68), those that had a family member or relative who passed away in the past six months (*n* = 9) and those who did not complete the questionnaire (*n* = 159) (Figure 1).

### 2.4. Ethical Considerations

This study was approved included online screens for obtaining informed consent and a structured questionnaire by the institutional review board of G University (IRB No. 1044396-202003-HR-072-01), and in accordance with the Declaration of Helsinki. All participants were provided with a form that explained the background and purpose of the study, the survey content, benefits of participation in the study, confidentiality, the storage and destruction of data, consent to participate in the study, the right to withdraw from the study and information about the researcher. Thereafter, written consent was obtained, and the participants were offered a small gift as a token of gratitude.

### 2.5. Data Collection

The data collection was performed by an approved institution that had no research interest and complied with the code of ethics and research methods of the Korean Association for Survey Research and the Statistic Acts. This institution is the first research company in South Korea that is known by approximately 71% of the population. A mobile link was created to access the questionnaire, and evaluations were finalized through several tests. The links for the online survey and consent form were shared through a mobile device (e.g., smartphone) or the Internet. The reliability of the acquired data was secured through strict screening and response time checks. The data were collected online through a structured survey. Participants who wished to participate in the study after reading the research notice were given more information on the research before we obtained their consent online, after which the survey was delivered to them. The data were collected online through a structured survey; potential participants were informed of the purpose of the research, confidentiality and anonymity of data, and that they could withdraw from the research or refuse to participate at any time without consequence.

### 2.6. Instruments

The study employed a structured survey that consisted of 24 questions regarding the death of patients and QODD, seven questions on life-sustaining treatments, 14 questions on general characteristics of nurses, and 15 questions on person-centered care nursing.

#### 2.6.1. The QODD Korean Language Version 3.2

The QODD is a self-reporting tool developed for healthcare providers who care for terminally ill patients at hospitals or in ICUs until their death [10]. This tool, which was translated into Korean, has been verified for its validity and reliability [11] and consists of 20 items on four subfactors, including the experience of the terminally ill patient at death, the medical treatment during the hours of death, the experience of the moment of death, and the overall experience of nursing [12]. The QODD score for each patient was calculated by summing all valid ratings of the 20 designated items, dividing the total by the number of valid items, and multiplying the obtained value by 10. QODD scores ranged from 0 to 100, and a higher score indicated a better quality of death as perceived by nurses [12]. Cronbach’s alpha was 0.8 at the time of development and 0.95 in this study.

#### 2.6.2. Person-Centered Critical Care Nursing (PCCN)

To calculate the extent of PCCN, a tool whose validity and reliability was verified through ICU, nurses in Korea was used [13]. This tool was verified using exploratory factor analysis (EFA) and confirmative factor analysis (CFA) and consists of four factors: compassion, individuality, respect, and comfort [13]. A 5-point Likert scale is used to score its 15 items. Its Cronbach’s alpha was 0.84 at the time of development [13] and 0.8 in this study, in which the higher the score, the more was the person-centered care performed by the nurse.

### 2.7. Data Analysis

The collected data were analyzed using PASW Statistics 18.0. A *t*-test was conducted to examine the effects of the treatment administered during the hours of death (sedative treatment, suspension of life-sustaining treatment, CPR) on patients’ QODD. A *t*-test and ANOVA were conducted to investigate the relationship between the general characteristics of nurses and the patients’ QODD, with Scheffe’s test being conducted for a post hoc analysis. Pearson’s correlation coefficient was used to determine the correlation between the PCCN and patients’ QODD. A multiple regression analysis (enter mode) was conducted to examine the factors that affect the QODD of terminally ill patients in the ICU. Variables that showed significant differences in QOOD by univariate analysis (received sedative treatment, consulted with a doctor, received CPR within 48 h of death), or had a significant correlation with QOOD by Pearson’s correlation coefficient (PCCN) and age were included in the multiple regression analysis. Before the regression analysis, we have checked the multicollinearity between the variables. However, there were no issues with the multicollinearity of variables.

## 3. Results

### 3.1. QODD, PCCN, and Overall Quality of Death as Perceived by Nurses

The average QODD score was 39.12 ± 21.40. The items with the highest scores in the “patient’s experiences at the end of life” category were being touched or hugged by loved ones (5.58 ± 3.13) followed by having pain under control, while the item with the lowest score was being able to feed oneself (2.30 ± 3.16). The item with the highest score in the “medical care at the end of life” category was the experience of receiving mechanical ventilation (3.35 ± 2.51). For the “experience at the moment of death” category, the highest score was anyone being present at the moment of death (5.58 ± 3.01). The average PCCN score was 53.27 ± 7.83, and the overall quality of death score was 6.13 ± 2.44 out of 10 (Table 1).

### 3.2. QODD and PCCN Based on the Treatment Received during the Patient’s Hours of Death

Not receiving CPR led to a significantly higher QODD score (*p* = 0.001) compared to receiving CPR within 48 h of a patient’s death. Receiving sedatives during the patient’s stay in the ICU led to significantly high QODD (*p* = 0.013) and PCCN scores (*p* = 0.035). The suspension of life-sustaining treatment had no significant difference in the QODD and PCCN scores (*p* = 0.104 and *p* = 0.969, respectively). However, when the patient had discussed their desired end of life treatment with the doctor, their QODD and PCCN scores were significantly higher (*p* < 0.001 and *p* = 0.006, respectively) than when they did not discuss their desired treatment (Table 2). According to a post hoc test, when the patient had discussed their desired end-of-life treatment with the doctor, their QODD was significantly higher than when they did not discuss their desired treatment, or they did not know whether they discussed it or not (Table 2). In addition, there were no significant differences between QOOD and PCCN according to the reason for not suspending the life-sustaining treatments (Table 2).

### 3.3. QODD and PCCN Scores According to the General Characteristics of Nurses

Gender, duration of service, religion, and highest level of education had no significant effect on the QODD score (*p* = 0.55, *p* = 0.411, *p* = 0.372, and *p* = 0.695, respectively) or the PCCN score (*p* = 0.306, *p* = 0.627, *p* = 0.196, and *p* = 0.482, respectively) (Table 3). A total of 54.7% (*n* = 164) of the participants responded that they did not receive specialized training for caring for the terminally ill after graduation (Table 3), and there was no significant correlation between these types of training and the QODD (*p* = 0.274) or PCCN scores (*p* = 0.238) (Table 3).

### 3.4. The Correlations among QODD, PCCN, and Overall Quality of Death

The PCCN score and overall quality of death and dying score both had a significantly positive correlation with the QODD score (*p* < 0.001 and *p* < 0.001, respectively) (Table 4).

### 3.5. Factors Associated with QODD

To identify the factors affecting the QODD score, a multiple logistic regression analysis was performed. The assumptions of the regression analysis model were satisfied. The Durbin–Watson statistic was used to obtain the autocorrelation error value of 1.58. Thus, no autocorrelation was detected in the model. Multicollinearity was verified using the tolerance and VIF (variance inflation factor) values, and there were no issues with the multicollinearity of all variables. The regression model was statistically significant (F = 9.35, *p* < 0.001). QODD was high when the patients discussed their treatment during the moments of death with their doctors (*p* = 0.002), when CPR was not performed 48 h before the moment of death (*p* < 0.001), and when the PCCN score was high (*p* < 0.001). The adjusted determination coefficient of the model (Adj R^2^) was 0.16 (Table 5).

## 4. Discussion

This study aimed to investigate the factors affecting the quality of death of terminally ill patients in ICUs, as evaluated by nurses, as well as factors including the extent of person-centered care provided by nurses.

### 4.1. QOOD and PCCN Score of ICU Nurses

First, the QODD score was higher than that observed in a previous study, which investigated the QODD score of ICU doctors and nurses [12]. In contrast, the PCCN score in this study was lower than the score reported in a previous study on ICU nurses from a general hospital [13]. All PCCN scores in this study in the categories of compassion, individuality, respect, and comfort were lower than those of the previous studies [14]. In specific categories of the QODD, pain control and having someone besides the patient during the moments of death led to the highest QODD scores, similar to the findings of a previous study [12]. The previous studies were conducted among nurses in general tertiary hospitals located in major cities, whereas this study was conducted among randomly selected nurses across the country working in different types of hospitals. Hence, the differences in the severity of illness of patients in the ICU, patient characteristics, and the working process of the hospitals cannot be eliminated.

### 4.2. QODD and PCCN According to the Patient’s Treatment at the End of Life

Second, in this study, nurses recognized that not doing CPR within 48 h of death and administering a sedative while patients were in ICU increased QODD. These results are similar to those of a previous study conducted on ICU doctors and nurses [12], in which the QODD scores were significantly high if CPR was not performed within 24 h of the time of death; the QODD scores were also higher if sedatives had been administered within 24 h of the time of death than if sedatives had not been administered. There are restrictions on ensuring patient comfort in ICU, and specific methods may vary depending on the studies. However, the common emphasis in previous studies is that it is important to maintain the physical and mental comfort of the patient during the time of death and involve the patient and his or her family as soon as possible in this decision-making for treatment at the end of life [15,16].

Considering that, in Korea, wherein comfort during hours of death and lack of physical pain are important elements contributing to a good death [4], it can be inferred that ICU nurses also consider these two elements to be important. However, the QODD score provided by nurses in Korea is significantly lower than that provided by those in other countries [17,18,19]. The variations in culture and medical environment may play a role in producing these differences, but they also reflect the low-quality of death experienced by ICU patients in Korea. In early 2018, the law of “well-dying”, which provides the right to make decisions on life-sustaining treatments for terminally ill patients, was finally enacted in Korea. Currently, there are only a few situations where healthcare providers can put this “well-dying” legislation into action; nonetheless, it is important to develop different strategies to improve the quality of death of terminally ill patients.

The QODD and PCCN scores were higher when the patients consulted with their doctors regarding their treatment during their time of death than when the patients did not consult, or it was unknown whether consultation was carried out. Interactions with healthcare providers are also an important attribute in defining a good death [4], and it is encouraging that nurses also feel this way. In a previous study, patients who received palliative care team (PCT) consultation were able to communicate their preferred treatment with healthcare providers, which resulted in a higher quality of dying score [20]. Thus, appropriate consultation and communication by patients regarding their preferred treatment with doctors significantly improved the patient’s quality of death. This study evaluated whether consultation was carried out between patients and doctors. However, the nurses’ personality, professionalism, and trusting relationship with the patient also contributed to ensuring a good quality death for both the patients and their families [4]. Therefore, nurses are critical to providing an environment in which patients can feel psychological and physical comfort at the time of death.

### 4.3. Improving PCCN Could Increase QOOD

Third, the QODD and PCCN scores were significantly and positively correlated. More specifically, the more proficient the nurse was in person-centered care, the higher the quality of the patient’s death. The attributes that define the quality of death are avoiding meaningless extension of life, as well as ensuring dignity, comfort, and interaction with healthcare providers [4]. The elements for evaluating person-centered care are compassion, individuality, respect, and comfort [13]. Therefore, the elements of person-centered care nursing coincide with QOOD. Professionals need to be well prepared in knowledge, skills, and attitudes to support the dignity of patients at the end of life [21]. However, 54.7% of the participants in this study reported not having received any specialized training for caring for individuals during their moments of death. Perceptions of the quality of death may vary according to culture, individual beliefs, and preferences. Additionally, due to the characteristics of the ICU, variables depending on the patient’s condition can have various effects. Therefore, although it is difficult to apply a common guideline to all patients, a strategy is essential to enhance the medical staff’s awareness of the importance of person-centered care and to develop the staff’s competencies to respect and empathize with patients in end-of-life care.

### 4.4. Consultation with Patients on the End-of-Life Treatment Issue Could Increase QOOD

Fourth, a regression analysis was conducted to investigate the factors affecting the quality of death. The results indicate that the quality of death improved when the patient consulted more frequently with the doctor about their preferred method of treatment, CPR was not performed within 48 h of death, and enhanced quality of person-centered care was provided. The agreement of the patient to not receive life-sustaining treatments did not have a significant effect; rather, it was consultation with the doctor regarding the patient’s preferred treatment that had a significant effect on the quality of death.

In most cases, the consultation about death is carried out after the patient is in a critical condition [3]; thus, it becomes difficult to consider and honor the wishes of the patient in Korea [3]. There have also been many cases where the referral to palliative care has been delayed [22,23], which has been reported to reduce the effect of the involvement of the PCT [24]. These results imply that consultation between the patient and healthcare providers should be carried out earlier. A previous study suggested that creating high-quality end of life care in ICUs can include aspects such as the patient being included when making decisions, the patient deciding the place of death, ensuring patient comfort, family presence in the ICU, visits by children, meeting family needs, preparing the family mentally, and asking whether the family prefers healthcare providers to be around when the patient passes away [25]. However, the multitude and types of preferences are likely to vary for each patient and their family; therefore, focusing on competent person-centered care by nurses, including empathic ability, may be crucial [26].

This study evaluates the QODD of ICU patients and analyzes the factors affecting it and provides implications and considerations in the end-of-life care of ICU patients. As emphasized by previous studies, it is necessary to ensure the physical and mental comfort of ICU patients rather than a meaningless extension of life during the time of death [4], so that patients and their family can be involved in decision-making for the patients’ dignified death. This study is meaningful in that it is the latest evidence confirming the direction for improving the QODD of ICU patients and the role of medical staff, including nurses, in it.

This study had a few limitations. First, the patient factors were reported based on the observation of the nurses, which may not be an accurate representation of the patients’ condition. Additional research is needed on using actual medical records to analyze patient factors and their effects. Second, the results of this study cannot be generalized to a larger population because only a selected number of nurses who voluntarily participated were given the surveys even though the survey was targeted at ICU nurses from across the country.

## 5. Conclusions

In this study, nurses recognized that the quality of death improved when the patient consulted more frequently with the doctor about their preferred method of treatment, CPR was not performed within 48 h of death, and enhanced quality of person-centered care was provided. To ensure patient dignity at the end of life, thereby helping patients experience a good quality death, more efforts should be made to improve nurses’ competency in providing person-centered care while ensuring that nurses can sympathize with and respect terminally ill patients.

## Figures and Tables

**Table 1:** QODD, PCCN, and overall quality of death as perceived by nurses (N = 300).

	Mean ± SD	Possible Score Range
**Total Quality of Dying and Death score**	39.12 ± 91.40	0–100
**Patient’s experiences at the end of life**		
Having pain under control	5.23 ± 2.88	0–10
Having control over what is going on around oneself	3.51 ± 5.98	0–10
Being able to feed oneself	2.30 ± 3.16	0–10
Being able to breathe comfortably	3.72 ± 7.39	0–10
Feeling at peace with dying	3.58 ± 5.32	0–10
Feeling unafraid of dying	4.05 ± 0.23	0–10
Being able to laugh and smile	2.81 ± 8.14	0–10
Keeping one’s dignity and self-respect	3.19 ± 1.39	0–10
Spending time with family, friends	4.43 ± 4.51	0–10
Spending time alone	3.31 ± 3.00	0–10
Being touched or hugged by loved ones	5.58 ± 5.13	0–10
Saying goodbye to loved ones	4.02 ± 0.59	0–10
Clearing up bad feelings	3.22 ± 2.02	0–10
Visits from a religious advisor	5.17 ± 1.07	0–10
Spiritual service before death	4.86 ± 8.19	0–10
**Medical care at the end of life**		
Experience in receiving mechanical ventilation	3.35 ± 3.51	0–10
Experience of receiving dialysis	3.11 ± 1.45	0–10
Discussion with doctors about wishes	3.34 ± 3.00	0–10
**Experience at the moment of death**		
Anyone present at the moment of death	5.58 ± 5.01	0–10
State at the moment of death	3.88 ± 8.89	0–10
**PCCN**	53.27 ± 0.83	15–75
Compassion	13.35 ± 0.84	4–20
Individuality	13.86 ± 0.20	4–20
Respect	14.68 ± 0.72	4–20
Comfort	11.38 ± 0.15	3–15
**Overall quality of death**	6.13 ± 1.44	0–10

QODD: quality of dying and death score, PCCN: person-centered critical care nursing, SD: standard deviation.

**Table 2:** QODD and PCCN, according to the patient’s treatment at the end of life (N = 300).

			QODD		PCCN	
		N (%)	Mean ± SD	t or F (*p*)	Mean ± SD	t or F (*p*)
Received CPR within 48 h of death	Yes	139 (46.3)	34.83 ± 0.83	−3.282 (0.001)	53.46 ± 0.90	0.398 (0.691)
	No	161 (53.7)	42.82 ± 1.25		53.10 ± 0.79	
Received sedative treatment while staying in ICU	Yes	247 (82.3)	40.51 ± 0.92	2.511 (0.013)	53.68 ± 0.85	2.115 (0.035)
	No	41 (13.7)	31.64 ± 0.98		50.88 ± 0.86	
Suspension of life-sustaining treatments	Yes	125 (47.2)	40.32 ± 0.32	1.631 (0.104)	52.68 ± 0.12	0.039 (0.969)
	No	140 (52.8)	36.16 ± 1.06		52.64 ± 0.29	
Patient consulted with doctor regarding the treatment at the end of life	Yes	97 (32.3)	46.83 ± 0.26 ^a^	10.111 (<0.001) a > b * a > c *	55.21 ± 0.78 ^a^	5.250 (0.006) a > b *
	No	179 (59.7)	35.81 ± 1.54 ^b^		52.10 ± 0.54 ^b^	
	Not sure	24 (8.0)	32.60 ± 6.29 ^c^		54.13 ± 0.81 ^c^	
The reason for not suspending the life-sustaining treatments	Ethically impossible	13 (9.3)	26.61 ± 9.81	1.318 (0.267)	49.15 ± 0.30	0.854 (0.493)
	Insufficient legal grounds	37 (26.4)	38.28 ± 3.07		53.65 ± 0.27	
	Refused by the family doctor	10 (7.1)	39.35 ± 3.61		53.70 ± 0.45	
	Refused by the family	77 (55.0)	34.99 ± 9.53		52.49 ± 0.89	
	Others	3 (2.1)	57.65 ± 6.84		55.67 ± 1.93	

QODD: quality of dying and death score; PCCN: person-centered critical care nursing; SD: standard deviation; CPR: cardiopulmonary resuscitation; ICU: intensive care unit; *: Scheffe’s test. ^a,b,c^: the value of mean ± SD.

**Table 3:** QODD and PCCN, according to the general characteristics of nurses (N = 300).

			QODD		PCCN	
		N (%)	Mean ± SD	t (*p*)	Mean ± SD	t (*p*)
Gender	Male	27 (9.0)	41.45 ± 0.65	0.593 (0.554)	54.74 ± 0.13	1.026 (0.306)
	Female	273 (91.0)	38.88 ± 1.49		53.12 ± 0.80	
Duration of service in ICU	6 months—less than 1 year	13 (4.3)	46.35 ± 8.06	0.892 (0.411)	51.46 ± 0.99	0.468 (0.627)
	1 year—less than 3 years	90 (30.0)	39.69 ± 0.13		53.67 ± 0.63	
	3 years or more	197 (65.7)	38.37 ± 2.14		53.20 ± 0.04	
Religion	Catholic	47 (15.7)	37.97 ± 9.97	1.047 (0.372)	54.91 ± 0.60	1.573 (0.196)
	Christian	82 (27.3)	42.24 ± 3.34		53.85 ± 0.32	
	Buddhist	11 (3.7)	43.28 ± 9.82		54.36 ± 0.20	
	None	160 (53.3)	37.56 ± 0.84		52.41 ± 0.68	
Education level	Bachelor’s	251 (83.7)	39.14 ± 1.35	0.365 (0.695)	53.08 ± 0.80	0.731 (0.482)
	Master’s	46 (15.3)	38.31 ± 2.33		53.98 ± 0.14	
	Doctorate	3 (1.0)	49.20 ± 0.20		57.67 ± 0.73	
Received specialized training for caring for the terminally ill after graduation	None	164 (54.7)	37.60 ± 1.55	1.302 (0.274)	52.19 ± 0.61	1.417 (0.238)
	One-time training	36 (12.0)	38.52 ± 2.75		54.11 ± 0.04	
	Less than 6 h of training	64 (21.3)	45.25 ± 0.85		55.11 ± 0.61	
	More than 6 h of training (*n* = 36, 12.0%)	36 (12.0)	39.90 ± 0.35		51.95 ± 0.47	

QODD: quality of dying and death score, PCCN: person-centered critical care nursing, SD: standard deviation.

**Table 4:** The correlations among QODD, PCCN and overall quality of death (N = 300).

Variables	QODD	PCCN	Overall Quality of Death and Dying
	r (*p*)		
QODD	1	0.296 (<0.001)	0.306 (<0.001)
PCCN	0.296 (<0.001)	1	0.131 (0.023)
Overall quality of death and dying	0.306 (<0.001)	0.131 (0.023)	1

QODD: quality of dying and death score, PCCN: person-centered critical care nursing.

**Table 5:** Factors associated with QODD (N = 300).

Variables	B	S.E	β	t	*p*	Adj R^2^	F (*p*)
Constant	12.036	10.828		1.112	0.267	0.16	9.345 (<0.001)
Age (years)	−0.273	0.215	−0.068	−1.265	0.207		
Received sedative treatment (No)	−5.447	3.361	−0.088	−1.621	0.106		
Received sedative treatment (Not sure)	−4.281	5.800	−0.039	−0.738	0.461		
Patient consulted with a doctor regarding the treatment at the end of life (No)	−8.020	2.541	−0.184	−3.156	0.002		
Patient consulted with a doctor regarding the treatment at the end of life (Not sure)	−12.805	4.480	−0.163	−2.859	0.005		
Received CPR within 48 h of death (No)	8.212	2.293	0.192	3.581	<0.001		
PCCN	0.714	0.148	0.261	4.829	<0.001		

QODD: quality of dying and death score, CPR: cardiopulmonary resuscitation, PCCN: person-centered critical care nursing, S.E: standard error.

## Data Availability

The data presented in this study are available on request from the corresponding author. The data are not publicly available due to protect the participants.
